# Gallic acid inhibits the release of ADAMTS4 in nucleus pulposus cells by inhibiting p65 phosphorylation and acetylation of the NF-κB signaling pathway

**DOI:** 10.18632/oncotarget.17509

**Published:** 2017-04-28

**Authors:** Yao Huang, Jian Chen, Tao Jiang, Zheng Zhou, Bin Lv, Guoyong Yin, Jin Fan

**Affiliations:** ^1^ Department of Orthopaedics, The First Affiliated Hospital of Nanjing Medical University, Nanjing 210000, P.R. China

**Keywords:** GA, ADAMTS4, NF-κB, p65, degeneration

## Abstract

This study investigated the inhibitory effect of gallic acid (GA) on the release of A Disintegrin and Metalloproteinase with Thrombospondin motifs 4 (ADAMTS4) through the regulation of the NF-κB signaling pathway, which is closely related to the matrix metalloproteinases in nucleus pulposus cells. Different concentrations of GA were added to TNF-α-induced human nucleus pulposus cells (hNPCs) and intervertebral disc degeneration rat model. ADAMTS-4 expression increased both in the TNF-α-induced nucleus pulposus cells and intervertebral disc degeneration rat model. By contrast, the release of ADAMTS-4 was reduced, and the TNF-α-induced apoptosis of nucleus pulposus cells was significantly inhibited after addition of GA at different concentrations. Further study found that the levels of phosphorylated p65 (p-p65) was increased and the classical NF-κB signal pathway was activated after the nucleus pulposus cells were stimulated by TNF-α. Meanwhile, GA suppressed the p65 phosphorylation and inceased p65 deacetylation levels. As a consequence, GA can decrease the expression of ADAMTS-4 in nucleus pulposus cells by regulating the phosphorylation and acetylation of p65 in NF-κB signaling pathways.

## INTRODUCTION

Intervertebral disc degeneration is closely associated with intervertebral disc protrusion, spinal stenosis, and spinal degeneration [1, 2]. Despite not being fatal, this disease has a high incidence rate and significantly reduces the quality of life of affected patients and thus worsens their personal and social economic burden [3]. It has a slow and complex pathogenesis, in which factors play a crucial role. TNF-α and IL-1β are the two most important inflammatory factors, and their continued accumulation in the intervertebral disc increases the release of matrix metalloproteinases [4, 5]. The intervertebral disc consists of internal nucleus pulposus, peripheral anulus fibrosus, and upper and lower endplates. The nucleus pulposus is an essential structure that maintains the height and elasticity of the intervertebral disc. It is composed of nucleus pulposus cell and extracellular matrix. The former is similar to cartilage cells and can secrete type II collagen (Col II) and proteoglycan, and thus displays similar characteristics to those of chondrocytes. Col II is involved in the composition of the extracellular matrix skeleton, while proteoglycan maintains the moisture and elasticity of the intervertebral discs [6].

Metalloproteinases produced in the process of intervertebral disc degeneration accelerate intervertebral disc degeneration mainly by degrading Col II and proteoglycans [7]. The mechanisms of metalloproteinases, particularly MMP-3 and MMP-13 have been reported by several studies. Meanwhile, ADAMTS4 is an autocrine factor of the nucleus pulposus cells and particularly crucial of degrading proteoglycans in intervertebral disc degeneration [8]. Inflammatory factors such as TNF-α and IL-1β can activate the NF-κB signaling pathway in the nucleus pulposus cells and stimulate the transcription of the downstream ADAMTS4 through the phosphorylation of IκB and Rel A (p65), thereby increasing the secretion of ADAMTS4 [9].

Gallic acid (GA) is a class of polyphenols present in tea and grapes and is widely available in nature [10]. Its compounds are used as antioxidants in several food and pharmaceutical industries, because they exhibit certain properties, such as anti-inflammation, antitumor, antioxidation, and bacteria inhibition [11]. In our study, GA has been observed to have an anti-inflammatory effect on nucleus pulposus cells. Although GA cannot directly reduce the expression of ADAMTS4 mRNA in the nucleus pulposus cells, it can inhibit the TNF-α-related phosphorylation of p65 and increase its deacetylation. Thus, it can inhibit the TNF-α-induced activation of the p65 signaling pathway and reduce ADAMTS4 expression.

## RESULTS

### Cell phenotype identification

**Figure 1:**
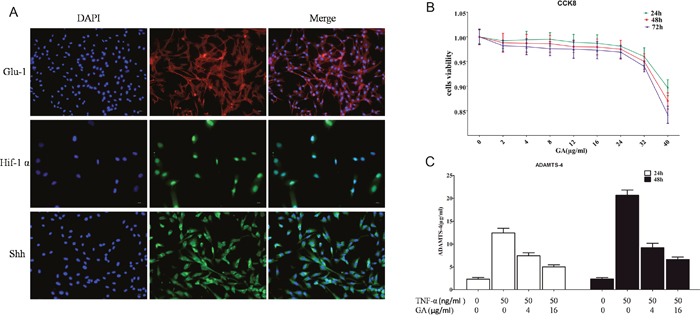
(A) Glu-1, Shh, and Hif-1α immunofluorescent staining Glu-1 fluorescence-labeled secondary antibody was Alexa Fluor 594, whereas Shh and Hif-1α fluorescence-labeled secondary antibody was Alexa Fluor 488. **(B)** CCK8 was used to analyze the toxicity of different GA concentrations on human nucleus pulposus cells cultured for 24, 48, and 72 h. **(C)** ELISA was used to analyze the effect of TNF-α and GA on supernatant ADAMTS4 secretion in human nucleus pulposus cells at 24 and 48 h.

The phenotypes of the human nucleus pulposus cells were identified first before purchase. The phenotypes were identified again after purchase. The results showed that the phenotypic markers of the nucleus pulposus cell were glucose transporter protein-1 (Glu-1), Sonic Hedgehog protein (Shh), and hypoxia inducible factor 1α (Hif-1α) protein, and the cells were consistent with the phenotypes characteristics of the human nucleus pulposus cells (Figure 1A).

### GA has low toxicity on nucleus pulposus cells

Studies have showed that GA is cytotoxic at high concentrations (Figure 1B). According to our results, GA had low toxicity against nucleus pulposus cells, and the CCK8 results showed that GA exceeding 32 μg/ml is toxic on the nucleus pulposus cells (2, 4, 8, 12, 16, 24, 32, and 40 μg/ml of GA was added successively).

### Inhibition of GA on TNF-α-induced secretion of ADAMTS4 in nucleus pulposus cells

**Figure 2:**
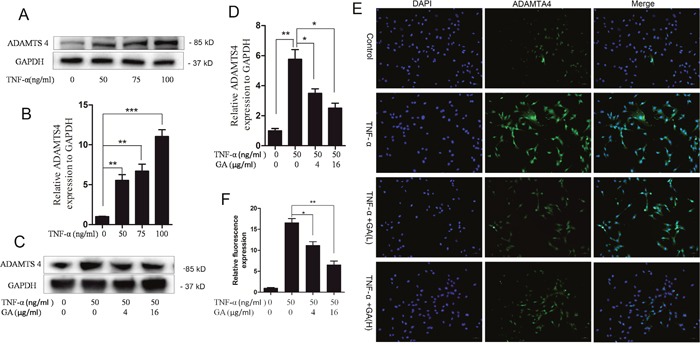
GA inhibits TNF-α-induced secretion of ADAMTS4 in hNPCs **(A)** Western blotting of ADAMTS4 in the human nucleus pulposus cells treated with different TNF-α concentrations for 24 h. **(B)** Comparison of Western blotting gray values among different TNF-α treatment groups. **(C)** Western blotting after human nucleus pulposus cells were treated with TNF-α and different GA concentrations for 24 h. **(D)** Comparison of Western blotting gray values among the groups treated with different TNF-α and GA concentrations. **(E)** Immunofluorescence of human nucleus pulposus cells treated with TNF-α and different GA concentrations for 24 h. **(F)** Comparison of immunofluorescence intensities among different treatment groups containing human nucleus pulposus cells. (*P < 0.05, **P < 0.01, ***P < 0.001)

**Figure 3:**
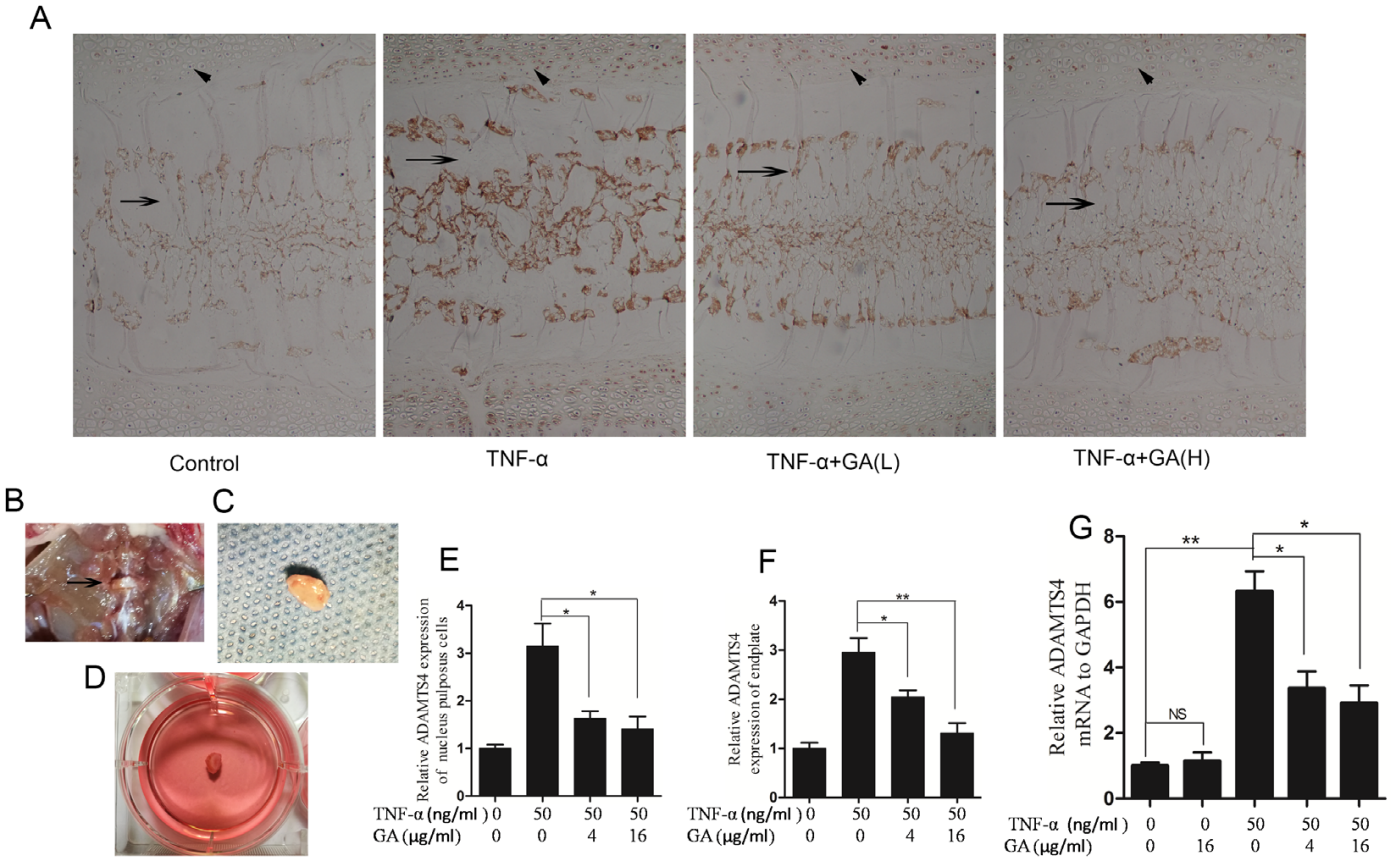
GA suppresses TNF-α-induced secretion of ADAMTS4 in rat intervertebral discs **(A)** Immunohistochemistry of rat intervertebral discs cultured for 3 days. Oblique arrow: endplates of intervertebral disc; horizontal arrow: nucleus pulposus of intervertebral disc. Original magnification, 100×. **(B)** Transabdominal approach dissect rat intervertebral disc. **(C)** Rat intervertebral disc was removed while retaining the endplates on both sides. **(D)** Intervertebral disc cultured in six-well culture dish. **(E)** Comparison among the immunohistochemical optical densities of nucleus pulposus of the groups treated with different TNF-α and GA concentrations. **(F)** Comparison among groups in terms of immunohistochemical optical density of rat intervertebral disc endplates. **(G)** Comparison among GA concentrations in terms of their influences on mRNA expression in human nucleus pulposus cells.

The supernatants of human nucleus pulposus cell culture media cultured at 24 and 48 h were collected for ELASA assay to determine whether GA influences the secretion of ADAMTS4 by nucleus pulposus cells. The results showed that the secretion of ADAMTS4 increased after the addition of TNF-α but decreased 24 and 48 h after GA addition (Figure 1C). We speculated that GA inhibited the secretion of ADAMTS4 in the nucleus pulposus cells, because it decreased ADAMTS4 concentrations in the nucleus pulposus cell culture media. The human nucleus pulposus cells were then subjected to Western blot and the results showed that ADAMTS4 secretion was significantly increased after TNF-α addition (Figure 2A, 2B) but apparently inhibited after adding GA with different concentrations (Figure 2C, 2D). We then observed the distribution of ADAMTS4 in human nucleus pulposus cells through immunofluorescence method and discovered that GA can inhibit the distribution of ADAMTS4 in cells (Figure 2E, 2F). In addition, after GA was added into the culture medium of intervertebral disc degeneration rat model, immunohistochemistry showed that GA had an inhibitory effect on the distribution of ADAMTS4 in rat nucleus pulposus cells (Figure 3A, 3E). ADAMTS4 secretion in the endplate cartilage cells on both sides of the rat intervertebral disc was similar to that of nucleus pulposus cells (Figure 3A, 3F). Given that endplate cells are cartilage cells, and nucleus pulposus cells are similar to cartilage cells, GA has the same effect on the cartilage cells. Thus, although GA were not able to directly reduce the expression of ADAMTS4 mRNA in the nucleus pulposus cells, it decreased of ADAMTS4 mRNA expression induced by TNF-α (Figure 3G).

### Inhibition of GA on the phosphorylation of p65

**Figure 4:**
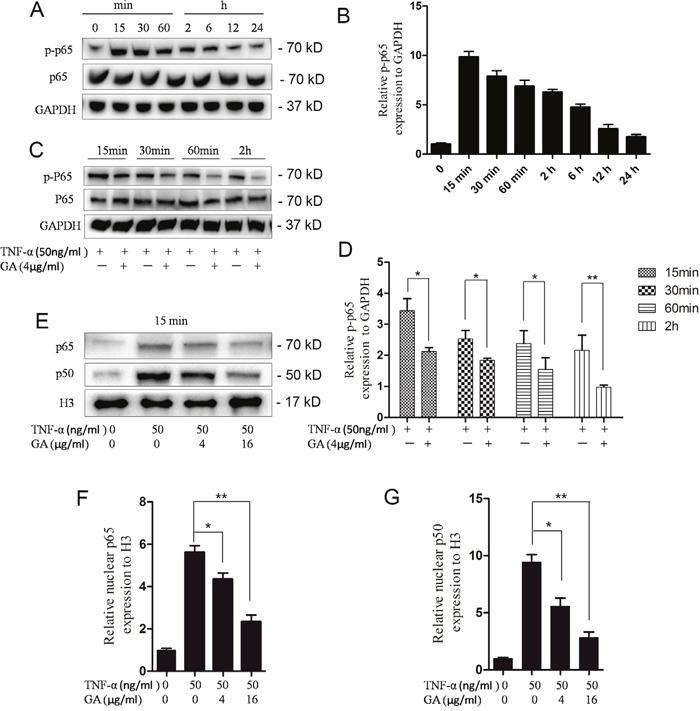
GA inhibits p65 phosphorylation and p65-p50 intranuclear **(A)** Western blotting of p65 phosphorylation rate at different time points after treatment of human nucleus pulposus cells (50 ng/ml). **(B)** Comparison of Western blotting gray values among TNF-α treatment groups. **(C)** Western blotting of phosphorylated p65 at four time points (15 min, 30 min, 60 min, and 2 h) after human nucleus pulposus cells were treated with TNF-α and GA. **(D)** Comparison among the Western blogging gray values of phosphorylated p65. **(E)** Western blotting of intranuclear p65 and p50 after human nucleus pulposus cells were treated with different concentrations of TNF-α and GA. (**F** and **G**) Comparison among the Western blogging gray values of intranuclear p65 and p50 of the groups.

In the nucleus pulposus cells, GA can antagonize TNF-α-induced ADAMTS4 secretion, in which TNF-α activates the NF-κB signaling pathway. Thus, we speculated that GA can inhibit the activation of the NF-κB signaling pathway. The p65 phosphorylation reached its peak level in the nucleus pulposus cells 15 min after TNF-α addition (Figure 4A, 4B). GA was added at four time points when the phosphorylation level of p65 was the highest (15 min, 30 min, 60 min, and 2 h). The phosphorylation level of p65 was inhibited in all time points (Figure 4C, 4D).

### Inhibition of GA on p65-p50 import into the nucleus

The import of the dimer p65–p50 into the nucleus was observed to verify the effect of inhibited p65 phosphorylation on the downstream pathways. Then, the p65 and p50 levels in the nucleus were examined. The results showed that the import was inhibited 15 min after GA and TNF-α additions (Figure 4E, 4F, 4G).

### GA decreased the acetylation level of p65 by inhibiting HAT activity

**Figure 5:**
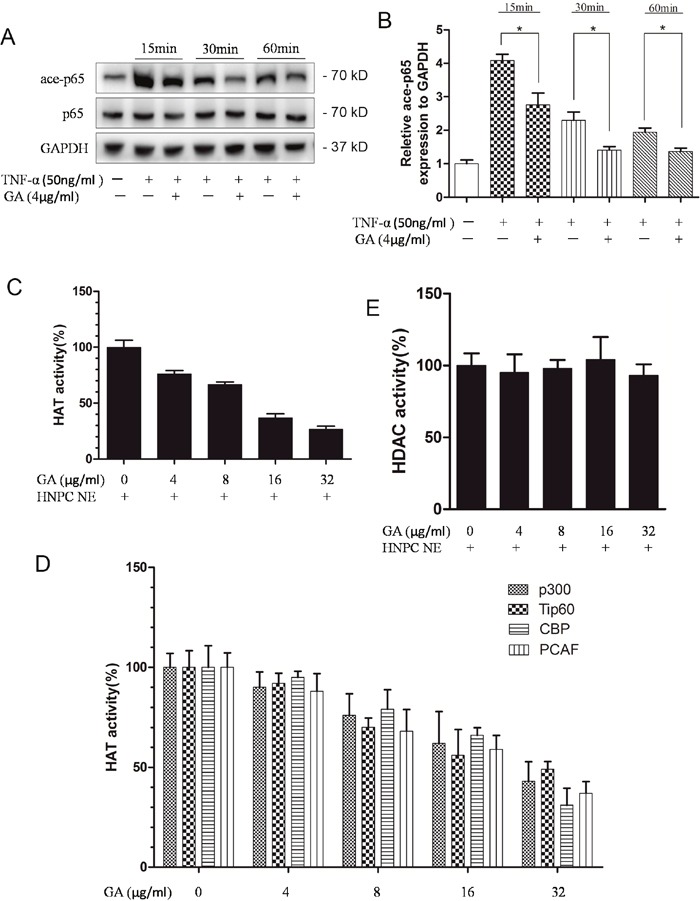
GA suppresses p65 acetylation **(A)** Western blotting of acetylated p65 at four time points (15 min, 30 min, 60 min, and 2 h) after human nucleus pulposus cells were treated with TNF-α and GA. **(B)** Comparison between the acetylated p65 Western blot gray values of the NF-α and GA treatments. **(C)** Effect of GA on HAT activity. **(D)** Effects of different GA concentrations on the activities of the four HATs (p300, Tip60, CBP, and PCAF). **(E)** Effect of GA on HDAC activity.

GA suppresses TNF-α-induced p65 phosphorylation and the transfer of p65–p50 into the nucleus. Thus, we speculated its impact on p65 acetylation. Compared with TNF-α group, the GA group had a decreased p65 acetylation level after 15 min, suggesting that GA suppressed p65 acetylation (Figure 5A, 5B). The acetylation level of P65 can be decreased by reducing HAT activity or increasing HDAC activity. Thus, we examined the influence of GA on HAT and HDAC activities. The results indicated that GA cannot activate HDAC (Figure 5E). We then tested several known HATs and found that HAT activity showed a decreasing trend at increasing GA concentration. Therefore, the deacetylation of p65 probably increased, because GA inhibited HAT activity (Figure 5C, 5D).

### Anti-apoptosis effect of GA on nucleus pulposus cells

**Figure 6:**
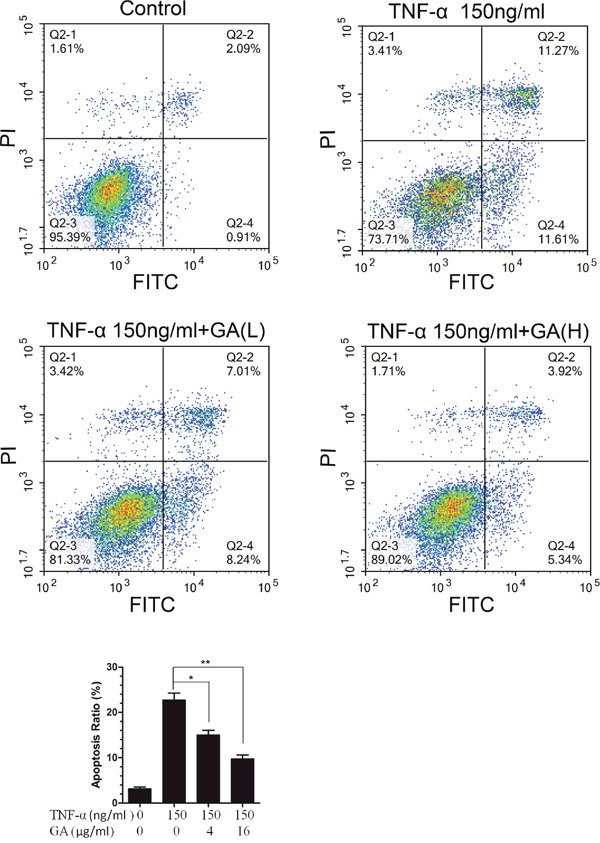
GA resists TNF-α-induced apoptosis in hNPCs **(A)** The hNPCs (1.0 × 105 cells/mL) were stimulated with or without GA(4 and 16 μg/ml) or TNF-α for 48 h in a 6-cm culture plate. Cells were collected and stained with FITC and PI and then examined by flow cytometry. **(B)** FACS histogram analuysis.

GA possesses cytotoxicity at high concentrations while TNF-α can induce cell apoptosis. Our results showed that GA antagonized the TNF-α-induced activation of NF-κB signaling pathway. Thus, the effect of GA on the apoptosis of nucleus pulposus cells were observed. The results indicated that 4 and 16 μg/ml GA antagonized TNF-α-induced apoptosis of nucleus pulposus cells when the TNF-α level was 150 ng/ml (Figure 6).

## DISCUSSION

The present study is the first to prove that GA can effectively inhibit ADAMTS4 secretion in the nucleus pulposus cells. Although ADAMTS4 plays an important role in the degeneration and senescence of intervertebral discs, studies on the effects of ADAMTS4 in the intervertebral disc are few. Thus, inhibiting the release of ADAMTS4 is of great importance for intervertebral disc degeneration, because ADAMTS4 is an autocrine factor of the nucleus pulposus cells.

Inflammatory factors play a critical role in intervertebral disc degeneration [12]. Some researchers have demonstrated that inflammatory factors promotes the secretion of ADAMTS4 in the nucleus pulposus cells [9]. Thus, antagonizing or regulating the effect of inflammatory factors can affect ADAMTS4 secretion in the nucleus pulposus cells, thereby delaying intervertebral disc degeneration.

GA can be extracted from green tea polyphenols and has anti-inflammatory bioactivity. Therefore, the anti-inflammatory activity of GA in the degeneration of nucleus pulposus cells was investigated. In our study, the autocrine level of ADAMTS4 was decreased after GA was added in TNF-α-induced rat intervertebral disc and hNPCs culture medium. During intervertebral disc degeneration, the inflammatory factors activated the NF-κB signaling pathway, leading to the secretion of various types of matrix metalloproteinases [13, 14]. The ADAMTS4 in these metalloproteinase degraded the proteoglycans that were particularly critical for maintaining the physiological function of intervertebral discs [8, 9, 15]. Therefore, the inhibitory action of GA on ADAMTS4 autocrine in the nucleus pulposus cells must be further investigated.

NF-κB signaling pathway plays an important role in immune regulation, inflammation, stress reaction, embryonic development, growth control, and cell apoptosis. During intervertebral disc degeneration, inflammatory factors such as IL-1β and TNF-α stimulate the nucleus pulposus cells to activate IκB kinase, thereby degrading IκB protein after ubiquitination and phosphorylation and exposing the NF-κB dimers, particularly dimer p65–p50. Then, p65 was phosphorylated and p65-p50 was imported into the nucleus to target the corresponding sequence of DNA according to the nuclear localization site of p50 [16]. We found that GA could inhibit p65 phosphorylation and suppress the import of p65–p50 into the nucleus which indicated that GA inhibited the activation of NF-κB signaling pathway. Dimer p65–p50 activated the transcription factors after entering the nucleus, and p65 was acetylated by histone acetyltransferase (HATs) in the nucleus at the same time. Acetylated p65 inhibited its ability to bind to IκBα, and led to the continous activiting of NF-κB signaling pathway. However, p65 was deacetylated by the histone deacetylase (HDAC) in the nucleus, strengthening its binding ability to IκBα, and the complex was transported out of the nucleus by the nuclear transport protein (CRMI). These processes are reasonable in organisms, and effectively prevent the excessive activation of NF-κB signal [17–19]. We also examined the effect of GA on p65 acetylation level, and the results showed that the p65 acetylation level was decreased as a result of GA addition. We speculated that GA can enter into the cell nucleus and subsequently affects p65 acetylation. GA reduced the acetylation level of p65 by inhibiting HAT activity, but it did not affect HDAC. Basing on this result, we determined that GA inhibits increase in acetylation but has no effect on deacetylation. Therefore, p65 acetylation decreased, leading to increased p65 export from the nucleus. This finding also indicated that GA can effectively inhibit the constant activation of NF-kB signaling pathway in nucleus pulposus cells.

In literature, high-dose GA is toxic to organisms [20]. Thus, we established a safe dose of GA for nucleus pulposus cells through CCK8 at the beginning of the experiment and then determined the GA concentration at which nucleus pulposus cells apoptosis occurs. Our aim is not only to determine the inhibitory effect of GA on ADAMTS4 autocrine, in other words, to delay the degradation of proteoglycans in intervertebral discs, but also to explore the possibility of actually applying GA in organisms.

According to the results of this study, a safe dose of GA has a significant inhibitory effect on the ADAMTS4 autocrine of nucleus pulposus cells stimulated by IL-1β and TNF-α and does not promote cytotoxicity and apoptosis. Meanwhile, we determined that GA inhibits the autocrine of ADAMTS4 by restraining the activation of the NF-κB signaling pathway. Several studies have showed that high GA concentrations promote apoptosis, and TNF-α is an important pro-apoptotic factor. However, our research results showed that low GA concentration antagonized TNF-α-induced apoptosis in the nucleus pulposus cells, and GA regulates the NF-κB signaling pathways. Moreover, another interesting phenomenon in this study we found was that the secretion of ADAMTS4 in the endplates on both sides of rat intervertebral disc was also regulated by GA, indicating that GA also affects the cartilage cells. The discoveries actively promoted the study of ADAMTS4 secretion in articular cartilage cells. We believe that these results can provide insights into the regulation of intervertebral disc degeneration and contribute to the development of methods that can antagonize intervertebral disc degeneration.

However, the systemic suppression of NF-κB can damage the defensive response functions of the body to pathogens [21]. Although the target spot of GA is p65, systemic use of GA may potentially cause damages to other organisms except intervertebral disc. Exploring the mechanisms by which GA targets in the intervertebral disc is necessary. In addition, despite being the largest tissue, the intervertebral disc lacks vessels, and thus its blood supply is extremely low, especially in adults. The nutrition of its nucleus cells mainly relies on a small amount of vessels from the bilateral endplates and anulus fibrosus. Thus, the transport of GA into the nucleus pulposus remains challenging.

**Figure 7:**
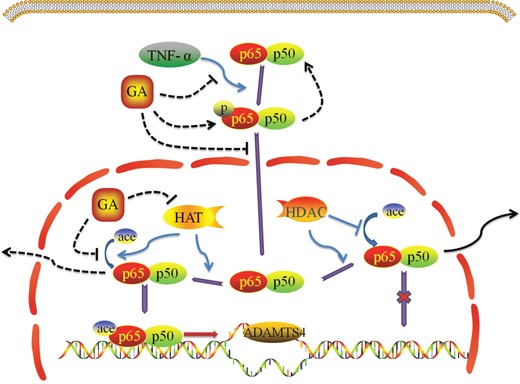
Effect of GA on TNF-α induced NF-κB signaling pathway

Taken together, the present study demonstrates that GA suppresses the p65 phosphorylation and acetylation of NF-κB signaling pathway thus inhibiting ADAMTS4 secretion of nucleus pulposus cells (Figure 7). This study adds a novel line of evidence in disc degeneration and may help in understanding disc degeneration therapeutics.

## MATERIALS AND METHODS

### Animals

The Sprague–Dawley rats were purchased from the Model Nanjing Animal Research Center of Nanjing University. All the procedures performed in this study complied with the ethical requirements of The First Affiliated Hospital with Nanjing Medical University.

The rat model of intervertebral disc degeneration was constructed (Figure 3B, 3C, 3D), and the secretion of ADAMTS4 in rat intervertebral disc was assayed after GA was added into the media [22].

### Cell culture, treatment of nucleus pulposus cells and intervertebral disc with TNF-α and GA

Human nucleus pulposus cells (ScienCell 4800, USA) and cell-specific media (ScienCell 4801, USA) were purchased from ScienCell. The cells were cultured at 37 °C/5% CO_2_, and then grown to 90% confluence. The cells within the five passages of subculture were then collected for the subsequent experiments. For the TNF-α-treated cells and intervertebral disc, the concentration of the culture medium was 50, 100, or 150 ng/ml. The concentrations for the GA-treated cells and intervertebral disc were 4 or 16 μg/ml, respectively.

### Cell counting kit 8 (CCK 8)

The CCK8 ptotocol was performed as described previously [23]. The medium was replaced with complete medium containing different concentrations of GA (0,2,4,8,12,16,24,32, and 40μg/mL). The complete medium was replaced with 100μL of serum-free medium containing 10% CCK8 dye after 1, 2, and 3 days, and cells were cultured for an additional 1h. The optical absorbance at 450 nm for each sample was measured using an absorbance microplate reader (ELx800 Absorbance Microplate Reader, Bio-Tek, USA).

### Enzyme-linked immunosorbent assay (ELISA)

ADAMTS4 release in the hNPCs was determined by enzyme-linked immunosorbent assay (ELISA) using a commercially available ELISA set (R&D Systems Inc., Minneapolis, MN and BD Biosciences, San Diego, CA). ELISA was performed according to the manufacturer's instructions. All samples and standards were measured in duplicate.

### Flow cytometry

Cells was assessed using an FITC Annexin V apoptosis Detection Kit I (BD Biosciences, Franklin Lakes, USA). Cells (1 ×10^6^/mL) were rinsed twice with PBS and then resuspended in binding buffer containing 5μL Annexin V-fluorescein isothiocyanate (FITC) and 5 μL propidium iodide (PI). The samples were then immediately analyzed using a flow cytometer (BD Biosciences, San Jose, CA, USA) and the data were analyzed using Flowjo software.

### Immunofluorescence and immunohistochemistry

For the immunofluorescence assays, hNPCs were cultured on a 24-pore plate. After fixed and incubated with 0.5% Triton X-100 (Sigma, Santa Clara, California, USA), the cells were blocked with goat serum for 1 h before the primary antibody (diluted by1:100; Abcam, Cambridge, UK) was applied for incubation at 4 °C overnight. The secondary antibody (diluted by 1:100; Jackson, West Grove, PA, USA) was applied and the cells were incubated for 1 h in a dark place. Finally, the nuclei were counterstained with DAPI for 15 min. The stained cells were photographed under a fluorescence microscope.

For the ADAMTS4 immunohistochemistry, The paraffin sections were de-waxed, dehydrated, and incubated overnight at 4 °C with anti-ADAMTS4 (diluted by1:100; Abcam, Cambridge, UK). After the primary antibody was removed, the secondary antibody (diluted by 1:100, Thermo, Waltham, Massachusetts, USA) was added; and, the sections after being incubated for 1 h at room temperature. The stained cells were developed with diaminobenzidine, counterstained with hematoxylin.

### RNA extraction and qRT-PCR

To determine the mRNA levels of ADAMTS4 and qRT-PCR was performed as described previously [24].

### Western blot analysis

Whole cell lysates were prepared as described previously [25]. Equal amounts (30 μg) of total proteins were subjected to sodium dodecylsulfate-polyacrylamide gel electrophoresis (SDS-PAGE) separation, followed by immunoblotting analyses with specific antibodies including antibodies against ADAMTS4, p65, p50, p-p65, Glu-1, Hif-1α, Shh, GAPDH and H3 (Abcam, Cambridge, UK). Anti–mouse immunoglobulin G (IgG) and anti–rabbit IgG antibody were purchased from Thermo (Waltham, Massachusetts, USA).

### HAT and HDAC activity assay

HNPCs nuclear extracts were prepared as described previously [26]. HAT activity and HDAC activity assays were done using nuclear extracts following the protocols of the manufacturer (BioVision Biotechnology). For HAT activity assays, immunoprecipitations were done using anti-p300, anti-Tip60, anti-CBP, and anti-PCAF (Abcam, Cambridge, UK) with hNPCs nuclear extracts. Precleared nuclear extract was incubated with antibodies overnight with Protein G dynabeads (Thermo, Waltham, Massachusetts, USA) at 4°C. All samples were counted with a multipurpose scintillation counter, LS 6500 (Beckman, California, USA).

### Statistical analysis

SPSS 19.0 was used for all statistical analysis. The measurements were presented as mean ± SD, and the data were analyzed using the Student t test and One-Way ANOVA test followed by Tukey's multiple comparison tests. All experiments were independently repeated 3 times and differences were statistically significant when P < 0.05.
